# Mobile Sensing Apps and Self-management of Mental Health During the COVID-19 Pandemic: Web-Based Survey

**DOI:** 10.2196/24180

**Published:** 2021-04-26

**Authors:** Banuchitra Suruliraj, Kitti Bessenyei, Alexa Bagnell, Patrick McGrath, Lori Wozney, Rita Orji, Sandra Meier

**Affiliations:** 1 Faculty of Computer Science Dalhousie University Halifax, NS Canada; 2 Department of Psychiatry Dalhousie University Halifax, NS Canada; 3 Nova Scotia Health Authority Halifax, NS Canada

**Keywords:** app, awareness, behavior, COVID-19, helpfulness, mobile health, mobile sensing, self-management, sensing, web-based survey

## Abstract

**Background:**

During the COVID-19 pandemic, people had to adapt their daily life routines to the currently implemented public health measures, which is likely to have resulted in a lack of in-person social interactions, physical activity, or sleep. Such changes can have a significant impact on mental health. Mobile sensing apps can passively record the daily life routines of people, thus making them aware of maladaptive behavioral adjustments to the pandemic.

**Objective:**

This study aimed to explore the views of people on mobile sensing apps that passively record behaviors and their potential to increase awareness and helpfulness for self-managing mental health during the pandemic.

**Methods:**

We conducted an anonymous web-based survey including people with and those without mental disorders, asking them to rate the helpfulness of mobile sensing apps for the self-management of mental health during the COVID-19 pandemic. The survey was conducted in May 2020.

**Results:**

The majority of participants, particularly those with a mental disorder (n=106/148, 72%), perceived mobile sensing apps as very or extremely helpful for managing their mental health by becoming aware of maladaptive behaviors. The perceived helpfulness of mobile sensing apps was also higher among people who experienced a stronger health impact of the COVID-19 pandemic (*β*=.24; 95% CI 0.16-0.33; *P*<.001), had a better understanding of technology (*β*=.17; 95% CI 0.08-0.25; *P*<.001), and had a higher education (*β*=.1; 95% CI 0.02-0.19; *P*=.02).

**Conclusions:**

Our findings highlight the potential of mobile sensing apps to assist in mental health care during the pandemic.

## Introduction

COVID-19 has currently affected over 213 countries [1]. In the absence of vaccines and antivirals, the remarkable speed and global spread of COVID-19 could so far only be reduced by rigorous implementation of traditional public health measures [2], such as quarantine and physical distancing. People have had to adapt their daily life routines to the currently implemented public health measures, which is likely to have resulted in a lack of in-person social interactions, physical activity, or sleep. These factors are known to have a significant impact on mental health, especially among vulnerable populations such as individuals living with a mental disorder. Preliminary health reports describe the adverse effects of the pandemic and its countermeasures on a range of aspects of mental health, including higher rates of anxiety, depression, abuse, and self-harm [3].

The recent proliferation of mobile sensing apps offers novel opportunities to monitor people’s behavior, thus potentially holding great promise for the self-management of mental health during the COVID-19 pandemic. Based on their passively recorded mobile sensing data, people could become aware of how their behaviors changed during the pandemic. For example, based on the global positioning system, accelerometer, and phone usage data, people could infer whether they are socially isolated, sleeping poorly, physically inactive, or not leaving their homes [4]. Importantly, such self-monitoring via mobile sensing has been previously shown to successfully increase people’s self-awareness [5]. Self-awareness is theorized to reflect an automatic process by which people compare their current behaviors to their internalized standards and is the first step to self-regulation; that is, the adaption of people’s actual behavior to their idealized behavior [6]. Thus, during the pandemic, mobile sensing apps might increase people’s awareness of maladaptive behavioral changes, thereby potentially motivating them to engage in health-promoting behavior. Such self-management becomes especially important in a scenario in which a many of the health services and social infrastructures that normally bolster against mental health problems during emergencies have been withdrawn [7].

We hypothesize that mobile sensing apps can increase self-awareness and thereby have a potential for limiting the adverse consequences of the pandemic on mental health. To test this hypothesis, we conducted a web-based survey to explore whether mobile sensing apps are perceived as helpful tools by people with and those without mental disorders for self-managing their mental health during the COVID-19 pandemic by increasing awareness for potential maladaptive behaviors.

## Methods

### Recruitment

We chose Amazon Mechanical Turk (AMT) as our web-based platform as it would facilitate rapid, large-scale participant recruitment [8]. Importantly, AMT has become an increasingly accepted means of collecting responses from diverse participants [9]. We therefore ran an anonymous web-based survey from May 23 to June 7, 2020. Following AMT’s standard procedure, we advertised the study and the qualification criteria on the platform worldwide. Interested participants clicked the link and responded to the survey. The survey was created using Dalhousie University's online survey platform Opinio. Participants received financial compensation for responding to the survey questions, which required 20 minutes of their time on average. All participants provided fully informed consent on the web-based platform. From among all participants aged ≥18 years, those who provided incorrect responses to 5 attention check questions and those who provided incomplete responses were excluded.

### Survey

Participants rated their agreement with the statement, “a mobile phone–based tracking application for health and well-being will be helpful in a pandemic or crisis situation like COVID-19,” on a 5-point Likert scale ranging from 1=“not at all” to 5=“extremely.” Answers to this question defined our outcome of interest; that is, the perceived helpfulness of mobile sensing apps. The concept of mobile sensing apps was introduced through multiple examples of what type of sensors might be used in mobile sensing apps and what behavioral insights might be obtained from these sensor data. In particular, we asked participants to rate the likeability and comfort with different mobile sensing features (Multimedia Appendix 1). Such questions have been previously shown to successfully convey the concept of mobile sensing apps [10].

Participants provided further information on the predictors of perceived helpfulness such as basic demographics (age, gender, and education) and their mental health history (with responses of “yes,” “no,” or “prefer not to answer”). We also asked participants to rate their technology knowledge on a 5-point Likert scale ranging from 1=“poor” to 5=“excellent.” Finally, we asked participants to rate the extent to which the COVID-19 pandemic has impacted their overall health and well-being, on a 5-point Likert scale ranging from 1=“not at all” to 5=“extremely.” An overview of all questions is provided in Multimedia Appendix 1.

We set the type I error rate (Cronbach *α*) at .05. Power analysis indicated that a sample of at least 410 participants would be required to detect a moderate effect (Cohen *d*=0.5) of our predictors with a power of 0.95.

### Statistical Analysis

After testing for homoscedasticity (determined using the Breusch–Pagan test [11]) and multicollinearity (determined from the variance inflation factor [12]), we used a linear model with the perceived helpfulness of mobile sensing apps as the outcome of interest and age, gender, education, mental health history, health impact of the COVID-19 pandemic, and technology knowledge as independent predictors. Additionally, we explored potential mediating effects based on the Sobel test [13]. SPSS (version 25, SPSS Inc) was used for all data analyses, and significance was set at *P*<.05.

### Ethics

All study procedures comply with the ethical standards of the relevant national and institutional committees on human experimentation and with the tenets of the 2008 revision of the 1975 Helsinki Declaration. All procedures were approved by the research ethics board at Dalhousie University. Furthermore, this study complies with the General Data Protection Regulation.

## Results

Cleaning for incorrect and missing responses resulted in a survey sample of 474 participants, most of whom were from the United States (n=237, 50%) or India (n=175, 37%). Of them, 235 (50%) were aged 25-34 years, 170 (36%) were female, and 148 (31%) had a history of a mental disorders (Table 1).

**Figure 1:**
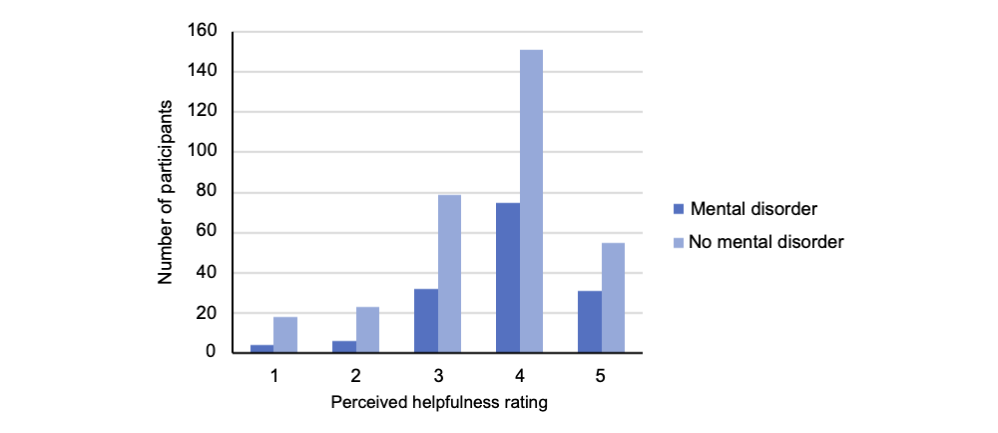
Perceived helpfulness ratings of mobile sensing apps during the COVID-19 pandemic.

The majority of our participants (n=312, 66%) perceived mobile sensing apps as “very” or “extremely” helpful for managing mental health during the COVID-19 pandemic. In total, 106 of the 148 (72%) participants with a history of a mental disorder found mobile sensing “very” or “extremely” helpful, whereas only 206 of 326 (63%) participants without a history of a mental disorder provided such responses (Figure 1). This difference was statistically significant, being controlled for age, sex, education, and technology knowledge (*β*=.12; 95% CI 0.03-0.21; *P*=.01), but fell short of significance after adjusting for the perceived health impact of the COVID-19 pandemic (*β*=.08; 95% CI –0.01 to 0.17; *P*=.06).

Specifically, participants with a history of a mental disorder reported experiencing a stronger health impact of the COVID-19 pandemic (*β*=.14; 95% CI 0.05-0.23; *P*=.002), which mediated the effect of a history of a mental disorder on the perceived helpfulness of mobile sensing (Sobel test, *P*=.01). Moreover, participants who experienced an even stronger health impact of the COVID-19 pandemic (*β*=.24; 95% CI 0.16-0.33; *P*<.001) had more technology knowledge (*β*=.17; 95% CI 0.08-0.25; *P*<.001), and those who had a higher education rated mobile sensing apps as more helpful (*β*=.10; 95% CI 0.02-0.19; *P*=.02); no differences were observed by age or gender (Table 2).

**Table 1:** Participant characteristics (N=474).

Characteristic		Participants
**Age (years), n**		
	18-24	90
	25-34	235
	35-44	75
	≥45	74
**Gender, n**		
	Female	170
	Male	304
**Education, n**		
	High school	51
	Bachelor's degree	336
	Master's degree	87
**Previously diagnosed with a mental disorder, n**		
	Yes	148
	No	324
Health impacted by the COVID-19 pandemic, mean (SD)		4.10 (0.70)
Have technology knowledge, mean (SD)		3.29 (1.14)

**Table 2:** Linear regression model of the perceived helpfulness of mobile sensing apps.

Parameter	*β*	SE	*P* value
Age	–0.07	0.05	.13
Gender	0.06	0.09	.18
Education	0.10	0.08	.02
Technology knowledge	0.17	0.06	<.001
History of a mental disorder	0.08	0.09	.06
Health impact of the COVID-19 pandemic	0.24	0.04	<.001

## Discussion

### Principal Findings

Our findings indicate that mobile sensing apps that passively track the daily life behaviors of people are perceived as very helpful tools for the self-management of mental health during the COVID-19 pandemic. People with and those without mental disorders considered mobile sensing apps as helpful for the self-management of their mental health during the pandemic, although people with mental disorders found them slightly more helpful. Importantly, people with mental disorders are reportedly at a higher risk of severe clinical outcomes of COVID-19 [14], and their mental health has deteriorated more during the pandemic compared to their counterparts without a history of mental disorders [15,16]. Concurrent with these previous reports, people with mental disorders reported a higher health impact of the COVID-19 pandemic in our study. Our results further indicate that this experience of a higher health impact of the COVID-19 pandemic is mediating the observed effect of the history of mental disorders on the perceived helpfulness of mobile sensing apps. In other words, people with mental disorders likely consider mobile sensing apps as more helpful as they struggle more with the impact of the COVID-19 pandemic than their counterparts without mental disorders.

Fringe events such as the COVID-19 pandemic provide opportunities to examine how mental health and behaviors deviate from baseline. Initial mobile sensing studies during the COVID-19 pandemic indicated that mobile sensing apps can identify maladaptive behaviors such as decreased physical activity and increased screen time [17,18]. In this regard, studies have consistently shown that physical activity, particularly aerobic activity, reduces self-reported mental health symptoms [19]. Other studies have shown that increased sedentary time, along with increased phone usage, is implicated in depression and anxiety [20]. Thus, from a health belief model [21] perspective, mobile sensing apps can help people become aware that it is possible to adopt behaviors in order to improve their mental health even during the pandemic; for example, reducing their sedentary and screen times. In addition, while mobile sensing apps can be beneficial for self-management, they can also provide cues to clinicians on how to best assist their patients during the pandemic, if patients agree to share their mobile sensing data with them. Importantly, while this study specifically explored whether mobile sensing apps could be useful to identify maladaptive changes in behavior during the pandemic, we believe that mobile sensing apps will be of value for the self-management of mental health symptoms beyond the context of the pandemic as well. For example, people might be struggling to revert to their prepandemic routines; thus, by increasing self-awareness, mobile sensing apps could help people revert to healthy routines more easily.

An important of caveat of mobile sensing apps is that there are some limitations to the interpretation of the recorded data. When people stay at home, they may not have their mobile phones with them at all times, which could lead to the overestimation of their sedentary time. Additionally, people may be preferentially accessing larger screens such as tablets or laptops; therefore, mobile phone usage may underestimate the total amount of screen time. Such shortcomings will need to be considered in the design of mobile sensing apps for mental health care.

While our results indicate that gender and age do not seem to impact the perceived helpfulness of mobile sensing apps for self-management of mental health during the pandemic, participants with high technology knowledge were more likely to find mobile sensing apps helpful. Accordingly, increasing the technology knowledge of users would be a crucial step for the acceptance and usability of mobile sensing apps for mental health care. Future studies should aim to further the current understanding of additional characteristics that might determine perceived helpfulness in order to enable efficient integration of mobile sensing apps in current mental health care models.

### Limitations

A considerable limitation of our survey is that our sample, though well-stratified and diverse, was not randomly recruited; people who have an interest in mobile sensing technologies might have been more likely to take part in this web-based survey. Our data might further be slightly biased by social desirability. However, we assume that such effects should only have been minimal, considering the anonymity of participants maintained throughout the survey. Nevertheless, our data suggest that a substantial number of people perceive mobile sensing apps as helpful tools for managing their mental health during the pandemic-related lockdown.

### Conclusions

Our findings indicate that the use of mobile sensing apps might have the potential to directly reduce the burden on the mental health care system during the COVID-19 pandemic by promoting better self-management. People with and those without mental disorders found mobile sensing apps as helpful to self-manage their mental health during the pandemic, although those with a mental disorder found such apps especially useful. By making users aware of maladaptive changes in their behaviors, mobile sensing apps can assist and motivate people to take better care of their mental health, preventing novel onsets or a worsening of mental disorders. Remote empowerment of people in mental health care must be considered especially valuable as standard ways of delivering care have been severely compromised during the COVID-19 pandemic. Finally, the ability of mobile sensing apps to increase self-awareness might have the potential to advance current health care models beyond the context of the current pandemic.

## Appendix

Multimedia Appendix 1 Survey Questions.

## Abbreviations

AMT: Amazon Mechanical Turk
