# Supplementary material for: Mobile Sensing Apps and Self-management of Mental Health During the COVID-19 Pandemic: Web-Based Survey
Source: JMIR Form Res. 2021 Apr 26;5(4):e24180. doi: 10.2196/24180 (PMC8078366; doi:10.2196/24180)
Supplement: Multimedia Appendix 1 [file formative_v5i4e24180_app1.docx]

Survey Questions

1. **What is your age?**

1. **What is your gender?**

1. **What is your highest level of education?**

1. **Based on your cultural practices, which country you are associated with?**

1. **Do you consider yourself as a technologically knowledgeable person?**

| Not at all | Not really | Somewhat | Moderate | Very much |
| --- | --- | --- | --- | --- |
| **** | **** | **** | **** | **** |

1. **Have you ever used a health application to track or manage stress, low mood, depression or anxiety?**

1. **Have you ever used a mobile phone-based tracking application for health and wellness such as fitness app, healthy eating, etc.?**

1. **Which of the below COVID-19 issues have a significant impact on you? (Select all that apply)**

Lockdown

Quarantine

Isolation

Travel ban

Financial issues

Other (Please specify)

1. **To what extent does COVID-19 impact your overall health and wellbeing?**

| Not at all | Slightly | Somewhat | Moderately | Very High |
| --- | --- | --- | --- | --- |
| **** | **** | **** | **** | **** |

1. **Do you use mobile phone? If yes what type?**

1. **Mobile phone-based tracking application for Health and Wellbeing - Overview**

*
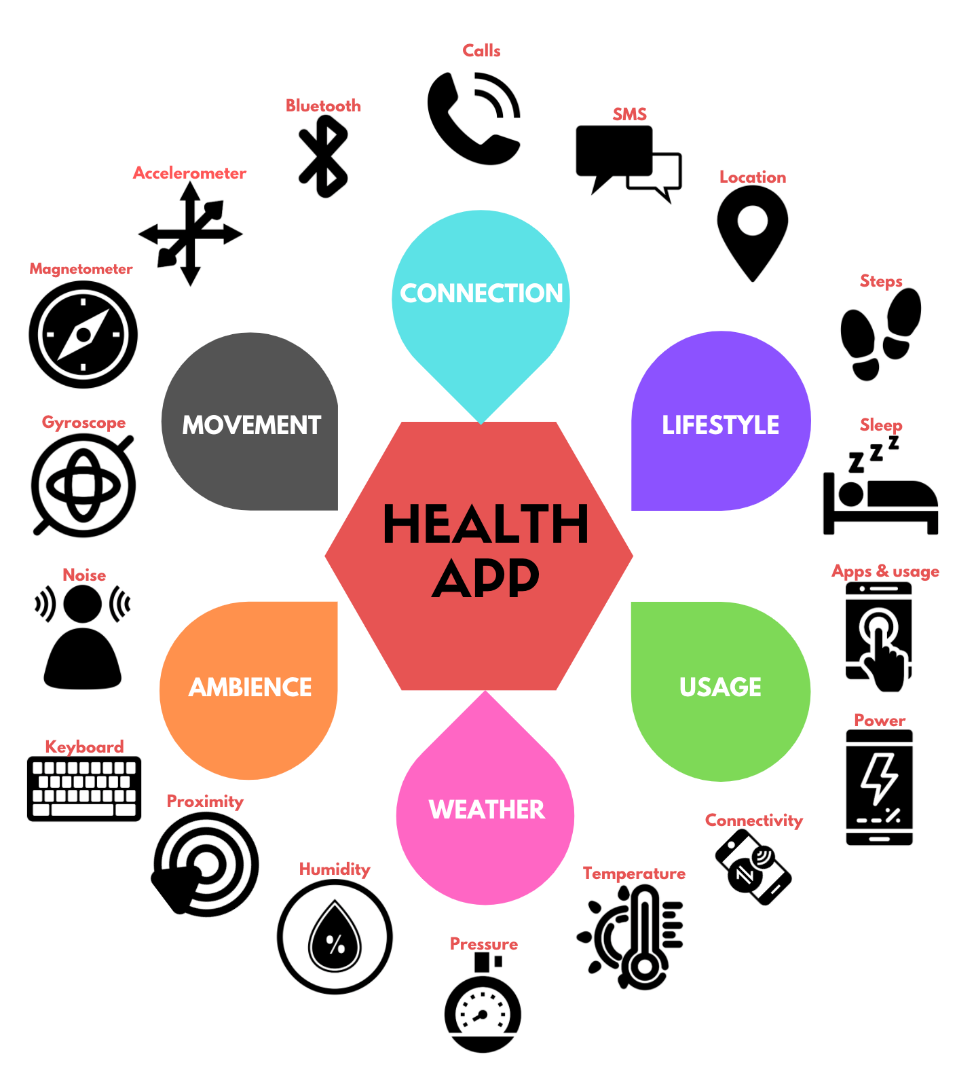
*

1. **The following questions are to understand your opinion about tracking data from your mobile phone with the purpose of helping you improve your health and wellbeing. Answer the questions below assuming a mobile application has the capabilities explained. Rate your response on a scale between 1 to 5, 1 being lowest and 5 being highest, to what extent would you like the following features?**

|  | 1 Not at all | 2 Slightly | 3 Moderately | 4 Very much | 5 Extremely |
| --- | --- | --- | --- | --- | --- |
| The app tracks your locations using GPS and notify you when your patterns are unusual. Example. Too much time spent outside or not going out at all | **** | **** | **** | **** | **** |
| The app tracks your communication pattern and notify you when your patterns are unusual. Example. Too much time spent in social media, or no communication at all | **** | **** | **** | **** | **** |
| The app tracks your mobile usage and predict potential health issues, App automatically predicts and notify you. With no data sharing. If you are paying attention pick option 3 | **** | **** | **** | **** | **** |
| The app tracks your health and sleep and notify you when your patterns are unusual. Example. Less/no sleep, missed exercise routines, less walk or too much sleep, too much exercise. | **** | **** | **** | **** | **** |
| The app tracks your environment and notify you when your patterns are unusual. Example. Too much noise, high temperature or less noise (always lonely) and freezing temperatures | **** | **** | **** | **** | **** |

1. **The following questions are to understand how comfortable you feel when data from your mobile phone is collected to predict your health issues. Answer the questions assuming a mobile application has the capabilities to collect data as explained below. Rate from 1 to 5 to state how much you are comfortable in the data collection feature, where 1 being lowest and 5 being highest.**

| **Mobile usage data tracking - All the time** | | | | | | **Mobile usage data tracking - Hourly average** | | | | | |
| --- | --- | --- | --- | --- | --- | --- | --- | --- | --- | --- | --- |
|  | 1 | 2 | 3 | 4 | 5 |  | 1 | 2 | 3 | 4 | 5 |
| Calls information: For every call, track data about call duration, type (incoming/outgoing). Does not collect the data about phone number or conversation |  |  |  |  |  | Calls information: Track hourly summary of number of calls and duration |  |  |  |  |  |
| SMS information: For every text message, track data about length, type (sent/received). Does not collect data on the content of messages |  |  |  |  |  | SMS information: Track hourly count of number of SMS sent/received, and count of typed keys |  |  |  |  |  |
| Bluetooth information: Track data about names of connected and nearby devices. Does not collect data on content sent/received |  |  |  |  |  | Bluetooth information: Track hourly count of number of Bluetooth devices nearby |  |  |  |  |  |
| Location information: Track data about every street you visit. Does not collect data on the building address |  |  |  |  |  | Location information: Track hourly summary of location changes in distance |  |  |  |  |  |
| Walking/running distance: Track data about number of meters walked/ran and time of the walk/run |  |  |  |  |  | Walking/running distance: Track hourly sum of number of meters walked |  |  |  |  |  |
| Health information: Please select Moderately if you are reading carefully |  |  |  |  |  | Health information: Please select Extremely if you are reading carefully |  |  |  |  |  |
| Light level: Track data about surrounding light levels all the time |  |  |  |  |  | Light level: Track hourly average of surrounding light level |  |  |  |  |  |
| Noise level: Track data about surrounding noise level all the time. Does not record audio |  |  |  |  |  | Noise level: Track hourly average of surrounding noise levels |  |  |  |  |  |
| Weather: Track weather conditions like temperature, pressure and humidity of the current place all the time |  |  |  |  |  | Weather information: Hourly average of temperature, pressure and humidity of the environment |  |  |  |  |  |
| Battery status: Track battery percentage all the time |  |  |  |  |  | Battery status: Track hourly average battery percentage |  |  |  |  |  |
| Music: Track data about name of the song for every played song |  |  |  |  |  | Music: Track hourly count of number of songs played |  |  |  |  |  |
| Movement: Track data about device movement, rotation and magnetic field all the time |  |  |  |  |  | Movement: Track hourly average of device movement, rotation and magnetic field |  |  |  |  |  |
| Screen time: Track data about app usage time for every app used on daily basis |  |  |  |  |  | Screen time: Track data about weekly average screen time |  |  |  |  |  |
| Notification: Track data about number of notifications received and name of the app on daily basis |  |  |  |  |  | Notifications: Track weekly count of number of notifications received and app names |  |  |  |  |  |
| Device information: Track data about mobile phone model and operating system |  |  |  |  |  | Device information: Track data about mobile phone, choose option Extremely if you are reading carefully |  |  |  |  |  |
| Pickups: Track data about count on number of times the phone is picked up from rest, and first opened app after pickup on daily basis |  |  |  |  |  | Pickups: Track weekly average of number of times the phone is picked from rest |  |  |  |  |  |

1. **To what extent are you knowledgeable about mobile tracking applications? Rate on a scale of 1-5 where 1 being poor and 5 being excellent.**

| 1 Not at all | 2 Slightly | 3 Moderately | 4 Very much | 5 Extremely |
| --- | --- | --- | --- | --- |
|  |  |  |  |  |

1. **Mobile phone-based tracking applications can be used to determine your health and wellbeing?**

| 1 Strongly disagree | 2 Disagree | 3 Neither agree nor disagree | 4 Agree | 5 Strongly agree |
| --- | --- | --- | --- | --- |
|  |  |  |  |  |

1. **Did you ever receive treatment for mental health illness in the past/present?**

1. **A mobile phone-based tracking application for health and wellbeing will be helpful in the pandemic/crisis situation like COVID-19**

| 1 Not at all | 2 Slightly | 3 Moderately | 4 Very | 5 Extremely |
| --- | --- | --- | --- | --- |
|  |  |  |  |  |
